# Metagenomics Approaches to Investigate the Neonatal Gut Microbiome

**DOI:** 10.3389/fped.2022.886627

**Published:** 2022-06-21

**Authors:** Zakia Boudar, Sofia Sehli, Sara El Janahi, Najib Al Idrissi, Salsabil Hamdi, Nouzha Dini, Hassan Brim, Saaïd Amzazi, Chakib Nejjari, Michele Lloyd-Puryear, Hassan Ghazal

**Affiliations:** ^1^Department of Fundamental Sciences, School of Medicine, Mohammed VI University of Health Sciences, Casablanca, Morocco; ^2^Department of Surgery, Faculty of Medicine, Mohammed VI University of Health Sciences (UM6SS), Casablanca, Morocco; ^3^Laboratory of Genomics and Bioinformatics, School of Pharmacy, Mohammed VI University of Health Sciences (UM6SS), Casablanca, Morocco; ^4^Mother and Child Department, Cheikh Khalifa International University Hospital, Mohammed VI University of Health Sciences (UM6SS), Casablanca, Morocco; ^5^Department of Pathology, Howard University, Washington, DC, United States; ^6^Laboratory of Human Pathologies Biology, Department of Biology, Faculty of Sciences, and Genomic Center of Human Pathologies, Faculty of Medicine and Pharmacy, Mohammed V University, Rabat, Morocco; ^7^Department of Epidemiology and Biostatistics, International School of Public Health, Mohammed VI University of Health Sciences, Casablanca, Morocco; ^8^Department of Epidemiology and Public Health, Faculty of Medicine, University Sidi Mohammed Ben Abdellah, Fez, Morocco; ^9^American College of Medical Genetics and Genomics, Bethesda, MD, United States; ^10^National Center for Scientific and Technical Research, Rabat, Morocco

**Keywords:** neonatal microbiota, microbiome, metagenomics, dysbiosis, colonization, womb sterile, delivery, breastfeeding

## Abstract

Early infancy is critical for the development of an infant's gut flora. Many factors can influence microbiota development during the pre- and postnatal periods, including maternal factors, antibiotic exposure, mode of delivery, dietary patterns, and feeding type. Therefore, investigating the connection between these variables and host and microbiome interactions in neonatal development would be of great interest. As the “unculturable” era of microbiome research gives way to an intrinsically multidisciplinary field, microbiome research has reaped the advantages of technological advancements in next-generation sequencing, particularly 16S rRNA gene amplicon and shotgun sequencing, which have considerably expanded our knowledge about gut microbiota development during early life. Using omics approaches to explore the neonatal microbiome may help to better understand the link between the microbiome and newborn diseases. Herein, we summarized the metagenomics methods and tools used to advance knowledge on the neonatal microbiome origin and evolution and how the microbiome shapes early and late individuals' lives for health and disease. The way to overcome limitations in neonatal microbiome studies will be discussed.

## Introduction

The human microbiome is a set of microorganisms (bacteria, viruses, protozoa, and fungi) residing in our body in different types of commensal relationships. The gut microbiota is the most important and consequential of them, with approximately 1,000 species (1) and 100 trillion microorganisms, which is ten times more than the number of eukaryotic cells that make up our body (2). It plays a role in regulating nutrient intake, intestinal motility, and metabolic and immunological development (3). However, how the newborn gut microbiota forms remains an open question. The combination of neonatal (gestational age, genetic history), maternal (delivery mode, diet), and environmental (e.g., antibiotic exposures) variables are considered to impact microbial colonization (4, 5), although the precise processes remain unknown. Several studies have shown that the infant gut microbiome can be influenced by different factors, leading to many diseases in the infant or child, including necrotizing enterocolitis, obesity, and inflammatory diseases (6, 7). Thus it is critical to understand the mechanism of microbiome colonization in newborns and how various factors may influence this process.

With the development, sophistication, and sensitivity of metagenomics and culturomics, identifying fecal microbes is improving our current knowledge about gut microbiota development, particularly during the early days' post-delivery (8, 9). Two methods have been widely used to examine microbial diversity: 16S rRNA gene amplicon sequencing and whole-genome shotgun metagenomics sequencing (WGS). WGS metagenomics sequencing allows the characterization of microbial whole genomes, while 16S rRNA gene amplicon gives a depth description of the diversity of certain taxonomic groups (10, 11).

The purpose of this review is to summarize and discuss the available amplicon-based and WGS metagenomics methodologies and tools used to profile the newborn microbiome and study the *in utero* transmission of microorganisms from mother to newborn.

## The Sterile Uterus and the *in utero* Colonization Hypothesis

Conflicting results from various studies have unsettled the notion of the *in utero* gut microbiome. The “sterile uterus” theory maintains that the embryo grows in a sterile environment *in utero*, and except for intrauterine infections during pregnancy, microbial colonization begins after birth (9, 12). However, this theory of the sterile uterus has been called into question by recent studies using both metagenomics and culture techniques that revealed the presence of microbial community in the meconium, placenta, blood umbilical cord, and amniotic fluid (13, 14). In addition, other researchers have reported the presence of a unique microbiota in the placenta and amniotic fluid, as well as in healthy women at the time of elective cesarean section, associated with low diversity and a prevalence of *Proteobacteria* (15). Likewise, other studies have discovered microbes in the umbilical cord blood and amniotic fluid in healthy women and those with pregnancy complications (16, 17). Several studies have found microorganisms in the meconium, lending credence to the *in utero* colonization of the baby's gut theory. *Staphylococcus* was reported as the most frequent bacterium in meconium samples, followed by *Enterobacteriaceae* family, *Enterococcus, Lactobacillus*, and *Bifidobacterium* genera (18).

In contrast, supporters of the sterile uterus theory attribute the identified microbial components to external contamination (19) because there is no indication of bacterial colony survival (20). Likewise, attempts to grow viable bacteria from placental samples in healthy pregnancies have thus far been unsuccessful (21). Perez-Muñoz et al. (12), on the other hand, support both the “sterile uterus” theory and “*in utero* colonization” as a methodology-associated artifact (22). They concluded that the data stating a sterile uterine environment was more robust. They claim that methodological techniques in which contamination is fairly easy are responsible for the observed *in utero* colonization (12). In well-controlled studies, oral, vaginal, and placental samples were compared to contaminated controls. They determined that while there are unique microbial profiles in vaginal and oral samples, they did not discover a distinct placental microbiome, supporting the sterile environment theory (23). Therefore, firm conclusions remain elusive, and further research in this field is still required.

## Impact of Pre and Post Natal Factors on the Neonatal Microbiome

**Figure 1:**
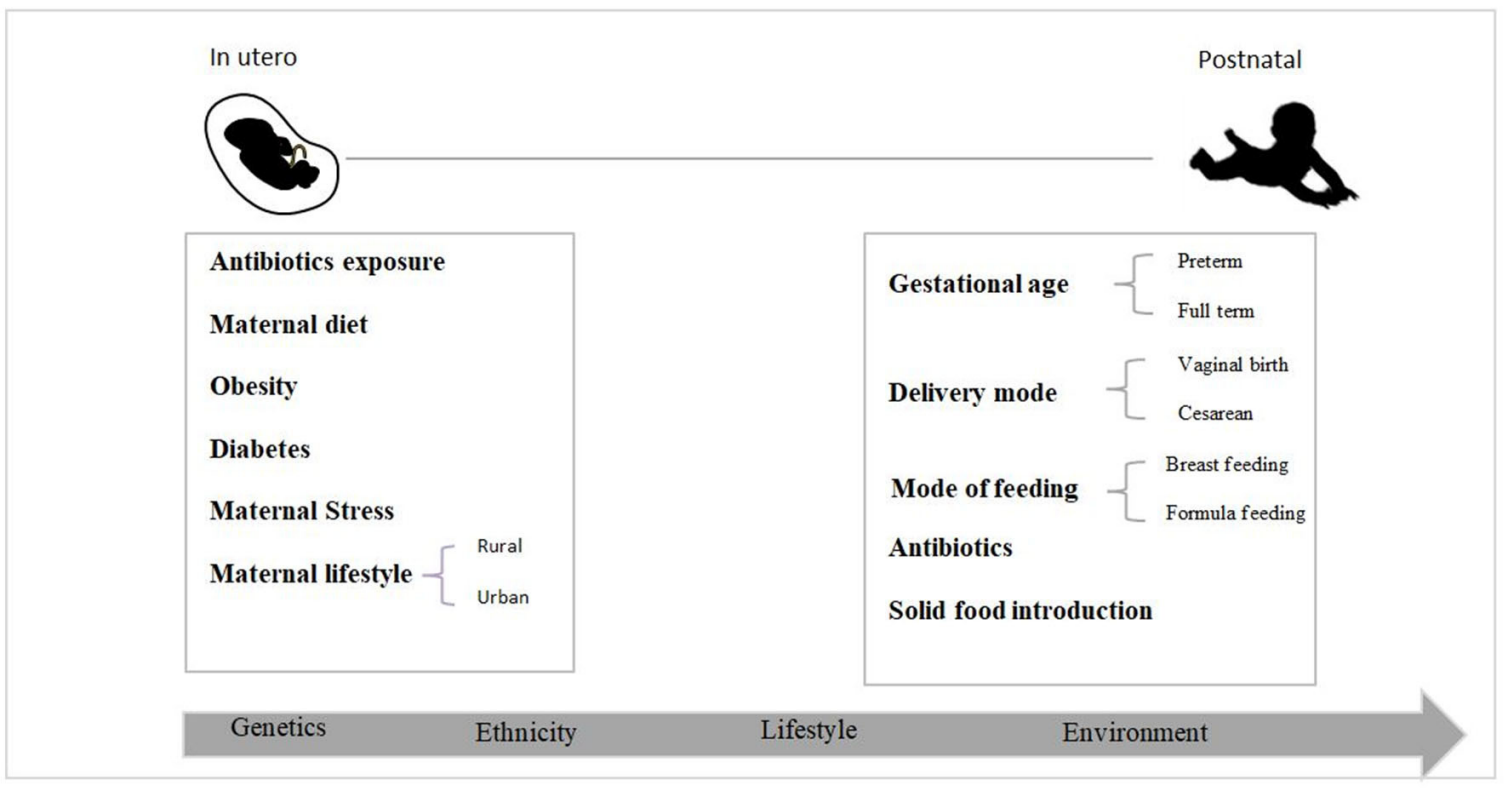
Early life factors that impact the newborn gut bacteria colonization process.

Several studies have shown that the gut microbiota has a role in the programming and development of the fetal immune system, metabolic programming, and preventing pathogen colonization of the gut, all of which have long-term consequences in infancy, early childhood, and adulthood (24–26). Colonization of the gut microbiome is a complicated process controlled by many variables (Figure 1) (27). Despite recent research on *in utero* colonization, the birth canal is still considered the baby's first bacterial encounter (28). The neonatal microbiota is dominated by *Enterobacteria, Escherichia*, and *Shigella* throughout the first few days (9, 29, 30). According to a new study (9), while the Firmicutes phylum dominates the meconium, Proteobacteria species dominate fecal samples from newborns throughout their 1st months of life. The newborn gut microbiota shows more significant fluctuations and minor variations in the early days of life. Nevertheless, as the infant ages, the bacterial communities increase in diversity and stability. It has been proven that by the age of two, the neonatal microbiota has stabilized to a level equivalent to that of adults (31). However, other scientists suggest that this process might take up to 5 years (32).

### Delivery Mode

It is well established that the delivery mode, either vaginal or cesarean section (CS), impacts gut microbiome colonization. *Lactobacillaceae* (Firmicutes) and *Proteobacteria* were initially characterized in vaginal-birth infants. Conversely, the gut of cesarean-delivered infants is dominated by *Streptococcaceae* and *Staphylococcaceae* (Firmicutes) (33, 34), with a low diversity during the first years of life (35). Interestingly, this difference in colonization disappears with age, and the microbiota of both vaginally and cesarean section-delivered infants become similar (36). Several studies have found that vaginal birth seeds have a more favorable and healthier microbiota than CS delivery seeds. Furthermore, it has been observed that the mode of delivery impacts the development of immunological responses that may lead to allergies and autoimmune diseases (37, 38). Extensive other studies demonstrated a link between cesarean delivery and a higher risk of asthma, obesity, and autoimmune disorders. However, more research needs to be done to provide a final answer to determine the relationship between mode of delivery and disease in later life (32).

### Gestational Age

Gestational age is an essential factor influencing the first colonization of an infant's gut. Several studies revealed differences in microbiota composition between term and preterm infants. The gut microbiota of full-term infants is generally dominated by *Bifidobacterium* and *Lactobacillus*, which are considered healthier bacteria (32, 39, 40), while preterm infants were found to have retarded colonization of *Bifidobacterium* and *Lactobacillus* in their early life. Instead, they are more likely to be colonized by potentially pathogenic bacteria, particularly *E. coli, Staphylococcus, Enterobacteriaceae*, and Bacteroides (41, 42). Premature infants are vulnerable to diseases including necrotizing enterocolitis (NEC) and sepsis, which are commonly caused by antimicrobial-resistant bacteria (38, 43, 44) due to their aberrant microbiota and immature gut immune systems. These diseases are rare in full-term babies, but they are severe and sometimes deadly in preterm babies. Moreover, gestational age has been revealed to influence milk composition, altering the metabolites that impact colonization, such as human milk oligosaccharides (HMOs) (15, 45).

### Antibiotics Exposure

Antibiotics are one of the most studied factors affecting microbiota, and they are the most prescribed treatment for infants. Antibiotic exposure and bacterial infections significantly affect microbiota composition in the postnatal and prenatal periods (46–48). Antibiotic treatment during pregnancy could affect the neonatal microbiota, leading to dysbiosis (48). Various studies have found a link between early gut microbiome dysbiosis and many diseases, such as asthma, immunological disorders (49), obesity, diabetes, and developmental disorders, such as autism, in later life. Early antibiotic exposure in infancy affects the composition and diversity of the infants' intestinal microbiota, with a reduction in *Bifidobacteria* and a marked increase in Proteobacteria. Furthermore, infants of mothers who received antibiotics before delivery showed the same microbiome changes as those seen in antibiotic-treated infants (48, 49). In addition, amoxicillin/clavulanic acid antibiotic medication was linked to a fourfold increased risk of newborn NEC (50).

### Nutrition

Nutrition during infant development plays a significant role in early microbiota colonization. Healthy full-term infants born through vaginal delivery and exclusively breastfed are thought to have the most beneficial gut microbiota composition. Human milk (HM) represents the optimal natural food (51) and contains a mix of nutrients, commensal bacteria, and functional groups such as oligosaccharides. HM also contains antimicrobials (such as lactoferrin) that can prevent the colonization of enteropathogens and stimulate the growth of *Bifidobacterium* (52), which may promote neonatal health by decreasing the risk of obesity and NEC and promoting mental development in preterm infants (53). Based on recent data, HM contains an average of 106 bacterial cells/ml (54), dominated by *Proteobacteria* and *Firmicutes* phyla, and a minor component of *Pseudomonas, Staphylococcus*, and *Streptococcus* genera (51, 53).

The exact mechanism through which bacteria reach the mammary glands and are excreted into breast milk is still debated (55). One hypothesis is that HM mainly contains bacteria derived from the mother's skin and/or the infant's mouth (56). The other hypothesis, called the “Enteromammary pathway”, postulates that some bacteria migrate from the maternal gastrointestinal tract to the mammary glands during late gestation and lactation (57). Formula-fed infants are codominated by *Bifidobacterium* and Bacteroides with a low percentage of *Escherichia coli* and *Clostridia* (52, 58). A high level of *Firmicutes* with a low level of *Bifidobacteria* has been associated with a predisposition to obesity (59).

The second shift in the microbial colonization process after breastfeeding, is the introduction of solid food. During this period, the microbiota is rapidly changing, and it is characterized by bacteria such as the *Ruminococcus, Blautia, Lachnospira*, and *Faecalibacterium* genera that can digest mucin and glycans and produce bioactive molecules such as short-chain fatty acids (SCFAs) (32).

### Genetics

Genetics also plays a role in microbial colonization, but the mechanisms are still poorly understood. Ethnicity has been recognized as one of the factors affecting neonatal microbial colonization, even in infants from the same geographic locations (60). Studies showed that the impact of ethnicity on newborn microbiota was visible 3 months after birth, and that was before the introduction of supplemental foods. A longitudinal study assessed the gut microbiota composition of 106 infants of three Asian ethnicities (Malay, Indian, and Chinese) who lived in the same geographical region (Singapore) and revealed that ethnic impacts were visible at 3 months post-birth and remained significant until 12 months in Chinese and Indian infants. The microbiota of Indian newborns was characterized by increased abundances of *Lactobacillus* and *Bifidobacterium*, while Bacteroides and *Akkermansia* were more abundant in Chinese infants (60). As a result of these ethnic variations in microbiota composition, human genetics may have a role in the establishment of gut microbiota.

Other studies on twins have discovered heritable bacteria in the gut microbiota, including the *Christensenellaceae* family and methanogenic *archaealarchaea* (60). MZ twins share more microorganisms than DZ twins or non-twin pairs, according to a study of ten healthy twins, five monozygotic (MZ) and five dizygotic (DZ), whose ages ranged from 0 to 6 years (61).

## Methods for Investigating the Human Microbiome

Prior to the microbiome era, bacterial characterization was primarily focused on identifying pathogenic species. However, the revelation of the importance of the microbiome-host interaction in human health has resulted in the creation of novel microbe investigation technologies. These so-called metagenomics techniques have helped researchers better understand the microbial diversity contained in a sample. This approach directly uses the genetic material existing in an environmental sample without the requirement for culture. Table 1 lists the most commonly used methodologies for studying the microbiome, as well as its benefits and limitations.

**Table 1:** Commonly used metagenomic techniques in microbiome analysis.

**Technique**	**Role**	**Advantage**	**Limitations**
Target amplification (16S rRNA gene Sequencing) (10, 11)	Identifying **taxa**	▪ Offer taxonomical information ▪ Quick analysis Cheaper than metagenomics	▪ Resolution limited to genus level ▪ PCR and primer biases ▪ False positive in low biomass samples
Shotgun metagenomics (62)	Presents all genome sequences found in a given sample	▪ Permit functional studies ▪ Taxonomic resolution ▪ to species or strain level	▪ Required more Bioinformatical analysis ▪ Functional analysis does not identify active genes ▪ Expensive
Metatranscriptomics (62)	Identifies and measures gut microbial mRNA, reveals which genes and pathways are active	▪ Gene expression and Viability data provided.	▪ Expensive and complex in sequencing Experimental issues (instability of RNA)
Metabolomics (62, 63)	Profiles the metabolites generated by the gut microbiome, defines biochemical pathways	▪ Great amount of data generated. ▪ Functional information	▪ Expensive techniques Complex analysis
Metaproteomics (62, 63)	identifies and quantifies proteins from microbial communities	▪ Provides more precise functional information	▪ Expensive techniques Complex analysis

The most common methods to explore the human microbiome are 16S rRNA gene amplicon sequencing and shotgun metagenomics sequencing (WGS) (10). The 16S rRNA genes consist of both highly conserved and variable regions used for taxonomic classification. At the same time, WGS presents all genome sequences found in a given sample (10, 11). Meta-omic studies have been increasingly used to study the gut microbiome community to better understand taxonomic classification, metabolic pathways, and the essential proteins and metabolites implicated in a specific host phenotype (64). One of these methods is metabolomics, which is used to profile the metabolites generated by the gut microbiome and define metabolites and biochemical pathways (62). Metatranscriptomics is another method for identifying and measuring gut microbial mRNA that reveals which genes and pathways are active and play essential roles in health and diseases (62). Another powerful approach to identifying and quantifying proteins from microbial communities is metaproteomics (63). Using a single omics approach to explore the gut microbiota has its own set of limitations (62), which can be addressed by combining various omics techniques. Consequently, researchers may better understand the relationship between the microbiome and diseases (65, 66).

There are several bioinformatic tools known to analyze 16S rRNA gene amplicon sequencing data, including QIIME 2 (67), Mothur (68), amplicon sequence variants (ASV)-based DADA2 (69), MED (70), and UNOISE (71). Operational taxonomic unit (OTU)-based methods such as QIIME eliminate sequencing errors by clustering the sequences in OTUs using a similarity threshold (usually 97%) (72, 73). On the other hand, ASV-based tools predict and correct sequencing errors (denoising) before forming clusters, allowing resolving sequences differing by a single nucleotide (69).

Previous studies have contrasted these two techniques and concluded that OTUs give poorer taxonomic resolution than ASVs and that choosing between the two can have an influence on alpha diversity estimations (73, 74). Nevertheless, data quality and PCR errors have a significant impact on ASV approaches, resulting in the loss of a significant quantity of relevant information. As a result, when the data quality isn't good enough, an OTU-based strategy is required (75).

Prodan et al. compared the most common bioinformatics pipelines for 16S rRNA gene amplicon sequencing (including MOTHUR, QIIME-UCLUST, USEARCH-UPARSE, DADA2, USEARCH-UNOISE3, Qiime2, and Deblur) (76). They found that DADA2 had the best resolution and sensitivity, but USEARCH-UNOISE3 had the best overall performance, combining high sensitivity with excellent specificity. However, to produce more robust data, research in this field needs to move toward improved methods (77). Another study by the Almeida group found that when comparing QIIME, QIIME 2, MAPseq, and Mothur, QIIME 2 was the best tool for composition prediction, while MAPseq was more precise, with few genera being misallocated (78).

The reference database utilized, in addition to selecting the appropriate bioinformatics tool, is a crucial aspect in ensuring the most significant classification performance. The Ribosomal Database Project (RDP), SILVA, and Greengenes are the most important 16S databases (79). However, SILVA is updated more regularly than Greengenes, which was last updated in May 2013. In addition, SILVA contains rRNA sequences of different species, including eukaryotic organisms, archaea, and bacterial species (79).

## Use of Metagenomic Techniques for Characterizing the Neonatal Gut Microbiome

The main metagenomic techniques and pipelines that have been used in neonatal microbiome studies are listed in Table 2.

**Table 2:** Metaomics technologies in neonatal microbiome studies (02/2021–09/2021).

**Approach**	**Study title**	**Samples**	**Bioinformatics tools**	**Outcomes**
16S rRNA gene sequencing	Fetal meconium does not have a detectable microbiota before birth (80)	Meconium (n=14) and infant stool (n=25)	**DADA2** (Taxonomic profiling)	▪ Fetal gut colonization of healthy term infants occurs at and after delivery, not before. ▪ Positive aerobic and anaerobic fetal meconium clinical cultures were detected as probable skin contaminants, most often *Staphylococcus epidermidis*, however, they were not discovered by sequencing in most samples.
16S rRNA gene sequencing Metabolomics	Distinct gut microbiota and metabolite profiles induced by delivery mode in healthy Chinese infants (81)	Stool samples from 60 infants	**Tax4Fun** (Microbial Functional profiling) **OSI/SMMS** (Metabolic profiling)	▪ Vaginally delivered infants had the highest abundance of *Bifidobacterium, Lactobacillus genera*, Bacteroides and Parabacteroides phyla, while the cesarean section delivered infants that had a high level of *Klebsiella* ▪ Vaginally delivered infants were associated with a high abundance of DL-norvaline and DL-citrulline. In contrast, cesarean section delivered infants were enriched in trans-vaccenic acid and cis-aconitic acid. ▪ Feces of vaginally delivered infants was positively correlated with tryptophan metabolism and pyruvate metabolism. However, feces of CS delivered infants was positively correlated with ABC transporters
Metagenomics	Human milk virome analysis: changing pattern regarding mode of delivery, birth weight, and lactational stage (82)	Transient HM sample (TMS; Postpartum (7–15 days) and mature HM samples (MMS; postpartum 45–90 days).	**QIIME** (Taxonomic profiling) **PRINSEQ-lite** (Filter and trim metagenomic data)	▪ HM virome may influence the composition of an infant's gut microbiome early in life, which might have short- and long-term health effects. ▪ Most prevalent virus family in the transitory HM of the regular spontaneous vaginal delivery group was Podoviridae. ▪ Myoviridae was predominant in both transient and mature HM in the premature group (all C-section), and Podoviridae was predominant in transient HM. At the same time, Siphoviridae and Herpesviridae were predominant in mature HM.
16S rRNA gene sequencing	Antibiotic treatments during infancy, changes in nasal microbiota, and asthma development: population-based cohort study (83)	Nasal samples from 697 children	**USEARCH** (Processing metagenomics data) **UPARSE** (Generating OTUs clusters)	▪ Exposure to ≥2 antibiotic treatments between the ages of 0 and 11 months was linked to an increased chance of developing asthma. ▪ Infants with more antibiotic treatments were more likely to have a profile with early *Moraxella* sparsity.
16S rRNA gene sequencing Metabolomics	Effects of antibiotic treatment and probiotics on the gut microbiome of 40 infants delivered before term by cesarean section analyzed by using 16S rRNA quantitative polymerase chain reaction sequencing (84)	Fecal samples of 40 premature infants delivered by cesarean section	**QIIME Mothur** (Taxonomic profiling) **Tax4Fun BugBase** (Measuring phenotypes in microbiome) **iPath** (Metabolics pathways)	▪ Antibiotics increase the prevalence of pathogenic bacteria while probiotics increase the prevalence of beneficial bacteria and the cellular community prokaryote function and contribute to the Bifidobacteria biofilm formation. ▪ Probiotics reduce the adverse effects of antibiotics on the composition and function of the gut microbiota ▪ Albumin was most factors influencing the composition of the gut microbiota at the genus level, and *Sphingomonas* was negatively correlated with Albumin.
16S rRNA gene sequencing	Maternal diet during pregnancy and intestinal markers are associated with early gut microbiota (85)	116 Maternal, neonatal fecal swabs	**DADA2**	▪ Maternal diet during gestation was associated with the diversity and richness of neonatal microbiota. ▪ Mothers having high lipids intake and consumers of SFA had lower Proteobacteria relative abundance in their microbiota. ▪ Total lipids were negatively associated with *Escherichia/Shigella* genus and positively linked to Firmicutes phylum, including genera from the Ruminococcaceae groups and the *Blautia, Roseburia, Rombustia*, and *Faecalibacterium* genera. These genera also showed a negative correlation with fiber and vegetable source proteins. ▪ Fat-related nutrient intake by mothers, including total lipids, SFA, and MUFA, showed enrichment in Firmicutes phylum genera and reduction in Proteobacteria phylum genera in the offspring microbiota
16S rRNA gene sequencing	Breastfeeding promotes early neonatal regulatory T-cell expansion and immune tolerance non-inherited maternal antigens (86)	Stool and blood samples of 38 term neonates born by cesarean section grouped according to feeding method (breast milk versus formula)	**QIIME2**	▪ Proportion of regulatory T cells (Tregs) increases at birth and 3 weeks of age. It is nearly 2 fold higher in exclusively breastfed neonates than those who only received formula milk. ▪ Breastfed neonates have a specific and Treg-dependent decrease in proliferative T-cell responses to non-inherited maternal antigens (NIMA), which is associated with a reduction in inflammatory cytokine production enrichment of short-chain fatty acid-producing taxa (*Veillonella* and *Gemella*) in stool samples of exclusively breastfed neonates.
16S rRNA gene sequencing	Transient effect of infant formula supplementation on the intestinal microbiota (87)	Stool and blood samples of 24 infants	**DADA2**	▪ Firmicutes, Proteobacteria, and Actinobacteria were the most frequent group found in all samples, bacterial genera *Bifidobacterium*, Bacteroides, and Parabacteroides had significantly higher relative abundance in vaginally delivered infants ▪ Supplementation caused transitory microbiota alterations, including increases in *Campylobacter, Dermabacter, Peptoniphilus, Prevotella*, and S24-7, as well as a decrease in *Eggerthella*. However, these differences diminished by the age of 6 months
16S rRNA gene sequencing	Maternal diet shapes the breast milk microbiota composition and diversity: impact of mode of delivery and antibiotics exposure (88)	120 Breast milk samples from healthy mothers	**DADA2**	▪ Maternal diet influences the composition and diversity of breast milk microbiota, with the most important contributions coming from dietary fiber and plant and animal protein intakes. ▪ Lower levels of *Lactobacillus, Bacteroides*, and *Sediminibacterium* genera were observed in Cluster II (high intake of animal proteins & lipids)/C-section/antibiotics exposure compared with the other groups
16S rRNA gene sequencing Metabolomics	Association of the birth mode of delivery with infant fecal microbiota, potential pathobionts, and short-chain fatty acids: a longitudinal study over the first year of life (89)	fecal Samples from 70 infants	**DADA2**	▪ CS infants had a higher abundance of the pathobionts *Clostridium neonatale* and *Clostridium perfringens* and a lower abundance of potentially beneficial *Bifidobacterium* and Bacteroides spp. ▪ a higher fecal butyrate concentration at 3 months.
16S rRNA gene sequencing	Influence of human milk on very preterms' gut microbiota and alkaline phosphatase activity (90)	117 preterm infants (≤32 gestational weeks).	**QIIME**	▪ HM was positively associated with beneficial bacteria, such as *Bifidobacterium, Bacteroides ovatus*, and *Akkermancia muciniphila*, as well as bacterial diversity. ▪ Neonates fed with HM during the first week of life had a higher abundance of *Bifidobacterium* content and fecal ALP activity on the 26^th^ postnatal day.
16S rRNA gene sequencing Metabolomics	The Effects of Different Modes of Delivery on the Structure and Predicted Function of Intestinal Microbiota in Neonates and Early Infants (91)	A stool sample from 82 healthy newborns (39 boys and 43 girls), .	**QIIME PICRUSt** (Functional profiling) (Pathway profiling) **KEGG**	▪ The genera *Bifidobacterium, Lactobacillus*, and *Bacteroides* were more prevalent in the vaginal delivery group than in the CS group, which exhibited a predominance of *Staphylococcus, Streptococcus*, and *Corynebacterium* in the 3-day-old infants' samples. ▪ In the samples from 30- to 42-days, *Bifidobacterium, Lactobacillus, Escherichia-Shigella*, and *Bacteroides* were the frequent genera present in the vaginal delivery group, while in the CS delivery group; the predominant genera *were Escherichia-Shigella, Bifidobacterium, Bacteroides*, and *Staphylococcus*. ▪ Predicted functions of the vaginal delivery group revealed higher metabolic and biodegradation rates of carbohydrates, vitamins, and xenobiotics than those in the CS group, which led to the stability of the microbiota.
16S rRNA gene sequencing	Maternal Vegetable and Fruit Consumption during Pregnancy and Its Effects on Infant Gut Microbiome (92)	39 infant stool samples were obtained at 2 months postpartum	**DADA2**	▪ The amount of fruits and vegetables consumed during pregnancy is linked to different alterations in the newborn gut microbiota at 2 months of age. ▪ Abundance of unhealthy infant gut microbiomes, such as *Erysipelatoclostridium*, Betaproteobacteria, and Lachnospiraceae, was negatively linked with higher maternal nutritional intake of fructose, dietary fiber, folic acid, and ascorbic acid.
16S rRNA gene sequencing	Gut microbiota development during infancy: Impact of introducing allergenic foods (93)	Fecal samples of 288 exclusively breast-fed infants	**DADA2**	▪ Exclusively breastfed infant at 3 months, gut microbiota was highly heterogeneous, forming three distinct groups: *Bifidobacterium*-rich, *Bacteroides*-rich, and *Escherichia/Shigella-*rich. ▪ Increased abundance of *Clostridium* sensu stricto at 3 months was linked to the presence of atopic dermatitis on examination at age 3 and 12 month. ▪ Introduction of allergenic solids promoted a significant increase in Shannon diversity and representation of particular microbes, such as Prevotellaceae and Proteobacteria (e.g., Escherichia/Shigella) as compared with infants exclusively breast-feed.

### 16S RRNA Gene Amplicon Sequencing

Previous studies using 16S rRNA gene sequencing to explore the gut microbiome have allowed the identification and functional characterization of microbial communities and highlighted the associations between microbial composition wellbeing and illness (6, 7, 49). Many studies have found that different factors, such as delivery mode, antibiotic exposure, and milk diet, significantly impact the formation of gut microbiota. In a birth-cohort study in Finland, 697 children showed that early exposure to two or more antibiotics was linked to a higher risk of asthma (83). Another study by Pan et al. examined the impact of the delivery mode on the structure and predicted function of intestinal microbiota in neonates and early infants in the Chinese population and discovered that the genera *Bifidobacterium, Lactobacillus*, and Bacteroides were more common in the vaginal delivery group than in the cesarean section group, which had a predominance of *Staphylococcus, Streptococcus*, and *Corynebacterium* in the 3-day-old infants' samples. Additionally, the Bacteroides level was lower in the cesarean section group than that in the vaginal birth group, suggesting that the latter is associated with an increased risk of obesity (91).

16S rRNA gene amplicon sequencing represents the principal tool for the characterization of bacteria in tissues with low bacterial biomass (i.e., placenta and meconium samples). This approach is limited by challenges associated with polymerase chain reaction (PCR)-based short read length sequencing, including GC bias, sequencing errors, and difficulty identifying OTUs (66, 67). Although it is unable to distinguish viable taxa, it continues to have technical difficulties in achieving species-level precision in taxonomic profiling.

### Shotgun Metagenomics

Shotgun metagenomics is the most informative technique for assessing taxonomic diversity present in a fecal sample (94). The findings of this analysis can be utilized to predict biological functions. The protein-coding sequences from the metagenomic readings are selected and compared to protein-coding sequences in a database to obtain functional profiling. This method may be used to provide a profile that describes the likely biological functions discovered in the sequenced metagenome (94, 95). For example, a recent study found that the HM virome may alter the makeup of an infant's gut microbiome early in infancy, thereby affecting both short- and long-term health (82).

Despite its numerous benefits, this technology has some limitations during DNA preparation, and post-analytical processing techniques suggest that this technology can yet be improved. The next point to consider is the high degree of expertise and high cost required to analyze such massive amounts of data (64, 69). Another technological limitation is that it is unable to make the difference between living and dead cells and thus unable to provide actual functional information (64, 69). Additionally, there are numerous incompletely annotated bacterial genomic sequences, as well as concerns regarding database correctness and coverage (96, 97).

Because metagenomics bioinformatics tools depend on the availability of annotated genomes, they are affected by reference sequence database restrictions. When evaluating metabolic potential, the absence of annotations for a large number of microbial species leads to a bias toward highly conserved pathways (housekeeping genes), even if there are major changes in taxonomic composition (64). Additionally, the lack of host DNA depletion kits makes metagenomics unreliable in tissues with low biomass such as placenta and meconium samples. Shotgun metagenomics (non-targeted sequencing) can quickly resolve species- and strain-level categorization, as well as reveal genome content, functional potential and partial genome assembly for organisms with low abundance. It is, however, still more costly than amplicon sequencing, is less tolerant of low biomass or contaminated materials, and requires more complicated and expensive analytic techniques.

### Metabolomics

Metabolomics studies on feces provide an essential analysis of the microbiome, including information on the host's metabolic profile, nutrition, and gut microbiota (62). This technique elucidates metabolites that mediate microbe-host interactions. In infancy, diet and mode of delivery have been found to have a significant connection with fecal metabolite composition (98). Human breast milk includes high levels of HMOs, which function as selective nutrients for particular bacterial groups (e.g., *Bifidobacterium*) in the production of SCFAs whose quantities change in relation to breastfeeding or formula diet. A recent study compared the gut microbiota samples of breastfed vs. formula-fed infants born by cesarean section (86). Breastfed neonates revealed a reduction in proliferative T-cell responses to non-inherited maternal antigens (NIMA) that was specific and Treg-dependent, as well as a decrease in inflammatory cytokine production (86). However, the fecal metabolome is thought to represent a functional output of the microbiome (99). Nevertheless, some of the metabolites will be shared by the gut microbiome and the host since feces include a combined metabolite output of both (62). However, because a large spectrum of metabolites is shared by the human host and intestinal microbes, metabolomics approaches are unable to differentiate them (100).

Although metabolomics is an excellent tool for investigating the role of bacteria in a variety of pathologies, including human intestinal disorders, and neurodegenerative diseases, it is limited by the inability to distinguish between host and bacterial metabolites (64), the complexity in associating the relative phylogenetic origin, and the lack of adequate reference databases (69, 70). Therefore, selecting suitable analytical methods and pipelines (equipment selection, sample processing, and statistical analysis) is a critical step in allowing relevant biological interpretation (64, 69, 70). Due to the large number and variety of metabolites discovered in a stool sample, as well as the heterogeneity of existing databases, data analysis may need manual integration of different databases by the user (100), which is a time-consuming and difficult phase that involves a significant chance of user-related mistakes.

## Challenges of Low Microbial Biomass Samples

Most microbiome research has focused on the gut, leading to the development of technologies that are better suited to samples with high microbial biomass (101). Samples from other body sites or, in comparison to the gut, have lower microbial biomass and are technically more difficult to explore. The main challenge with these samples is the amount of DNA, which comes from the environment. Microbial contamination has been presented as a real problem due to a lack of vigilance and understanding of technical problems while working with low microbial biomass samples (101–103). The publication of placental microbiota is an excellent illustration of this (101). This resulted in a false-positive finding, and unfortunately, other researchers have followed the same path.

Microbiome analysis has previously had problems due to a lack of controls. Initial research on the placental microbiota, for example, aimed to solve many issues about baby development and premature birth during pregnancy (13). Aagaard et al., on the other hand, did not take the essential procedures to monitor and reduce contamination. During the extractions, non-template controls were used, but only a fraction was sequenced. The sequences obtained in the negative controls were not compared to those found in the biological samples in any way. Furthermore, no environmental controls were collected or examined, preventing environmental contaminants from being detected.

Finally, the detection limit was not specified, prohibiting the researchers from determining whether a credible signal could be identified. Contaminant microbial species could not be reliably identified or analyzed in placenta samples, making it impossible to verify whether the placental microbial signature was genuinely endogenous. Lauder et al. (23) discovered that the microbial placenta profiles were similar to those of extraction blank control (EBC) and air samples, indicating that the placenta was likely sterile and lacked a varied microbial signature. Many other well-controlled investigations have now shown the absence of a placental microbiome (19–21). These studies emphasize the necessity of having solid processes and controls in place to avoid biased results.

As awareness of the aforementioned challenges of low microbial biomass sample examination, more researchers are now including controls in their data analysis. Nevertheless, this practice is still not standardized across the board, and researchers urgently need to learn from these past errors to improve microbiome research in the future, in particular for newborns. More researchers are increasingly integrating controls from sampling to data analysis as knowledge of the limitations of low microbial biomass sample inspection has increased. Nonetheless, this method is not uniform across the board, and researchers must learn from previous mistakes if they are to enhance microbiome research in the future.

## Recommendations for Avoiding Possible Contamination in Low-Biomass Samples

Common microbiome procedures are not optimal for low microbial biomass samples, even in today's common microbiome research. Low biomass laboratory and analytical techniques have improved as a result of recent research, but there is always a potential for improvement (103, 104). To prevent contamination and biases, greater knowledge of when and how they arise is needed. DNA contamination and biases can occur at any step during the sample preparation and analysis process. Therefore, it is recommended to include controls from the samples of the professionals who collect and process samples, the collection room, the laboratory environment, equipment, and reagents. Moreover, all sample collectors and laboratory technicians should wear clothes that cover exposed skin, such as gloves and face masks (102, 103). Additionally, ultraviolet radiation can be used to minimize reagent and equipment contamination (101). It is also recommended that low biomass samples should be sequenced at a higher depth to capture a sufficient number of unique sequences (105).

Several bioinformatic approaches have been developed to monitor and remove contaminated DNA that affects samples with low microbial biomass. First, as previously mentioned, the limit of detection can be achieved using positive and negative controls (101–103). Individual contaminated species may also be traced using their specific sequence from their source (i.e., equipment, reagents, environmental controls, etc.) and then eliminated or identified using publicly accessible software such as SourceTracker and Decontam (101, 106). While programs can be used to eliminate or track contaminants, they do not replace the need to monitor and investigate contaminants throughout the sampling and laboratory processes.

As the number of investigations into neonatal microbiome samples grows, it is critical that new protocols and methods are developed to reduce the impact of contamination and biases on these samples, as well as to fully understand the limitations when developing diagnostic tests based on the findings (101–103). To reduce some of these difficulties and better detect disease causes and consequences, new methods based on sensitive, high-throughput techniques that explore undiscovered microbes and microbial populations as a whole are necessary.

## Conclusion

With the advent of metagenomics, we can now define the structure and function of the microbiome community at the pre- and post-delivery stages with high precision. Due to the metagenomics' potential, dogmas such as the “sterile womb” hypothesis are being challenged by the discovery of microbes in previously sterile tissues. However, if these bacteria are rucked as real occurrences and not just experimental artifacts/contaminants, it is unknown if they colonize the embryo or are only present for the purpose of priming the fetal immune system.

Our review of maternal and environmental factors influencing intestinal microbiota highlights the need to focus on these aspects during pregnancy. The gut microbiome plays a significant role in human growth and development, as well as in the establishment of immunological responses, indicating that balanced gut microbiota is essential for good health. Emerging data suggesting early-life gut microbiome establishment as a protective factor against gut dysbiosis–related diseases later in life supports the need for targeted therapies to restore the gut microbiome in early-life.

To keep up with the latest sequencing technology and Metagenomic methods, new bioinformatics tools are constantly being developed and updated. The most difficult issue for the user is not being confused on offered alternatives. Indeed, comparing the outcomes obtained through several Bioinformatic tools remains the best strategy. Furthermore, using frequent comparison studies and reviews to help the user is beneficial and should be read and well analyzed before making a final decision on which tool to use. Therefore, to understand the neonatal microbiome and the newborn-health link, it is necessary to choose efficient computational tools and methodologies to analyze the neonatal microbiome.

## Future Directions and Perspectives

Even though microbiological techniques have advanced in the last years, certain aspects still need to be enhanced such as reducing contamination, which is a critical issue in microbiome studies, particularly for samples with low microbial mass, as well as the differences in sample collection, storage, DNA extraction protocols, sequencing methods, and bioinformatics tools, which could all be responsible for introducing biases into the results and variability between microbiome studies. Additionally, other sections of the infant gut microbiota composed of fungi and viruses require additional investigation. Furthermore, using fecal samples to study neonatal gut microbiota has limitations. It is not always representative of the gut microbiome and leaves out gut-adherent bacteria that affect colon epithelial physiology and functions.

Although dysbiosis has been associated with several diseases, such as inflammatory diseases, atopic diseases, and NEC, other conditions have also been linked to disruptions in the microbiome. Nevertheless, in most cases causal correlations have yet to be demonstrated. Moreover, is there a critical period of microbiome development that, if disturbed, leads to a disease state in cases where illness states have been connected to microbiome alterations? Are there some microorganisms that can protect you from diseases? What human host variables and/or environmental interactions are crucial for the development of a healthy newborn microbiome? Scientists working to determine the likely microbial etiologies of infant diseases must address these and other problems. By obtaining these responses, potential protective and therapeutic strategies can be created to modulate initial microbial colonization and decrease the risk of adverse health outcomes associated with unbalanced microbiota evolution.

## Author Contributions

HG, ZB, and ND contributed to conceptualization and writing. SS, SE, and NA contributed to writing. SH, HB, NA, ML-P, SA, and CN reviewed the manuscript. All authors read and approved the submitted version.

## Funding

The Article Processing Charge (APC) paid by Mohammed VI University of Health Sciences (UM6SS).

## Conflict of Interest

The authors declare that the research was conducted in the absence of any commercial or financial relationships that could be construed as a potential conflict of interest.

## Publisher's Note

All claims expressed in this article are solely those of the authors and do not necessarily represent those of their affiliated organizations, or those of the publisher, the editors and the reviewers. Any product that may be evaluated in this article, or claim that may be made by its manufacturer, is not guaranteed or endorsed by the publisher.

## References

[1] Qin J Li R Raes J Arumugam M Burgdorf KS Manichanh C. A human gut microbial gene catalogue established by metagenomic sequencing. Nature. (2010) 464:59–65. 10.1038/nature08821 20203603 PMC3779803

[2] Thursby E Juge N. Introduction to the human gut microbiota. Biochem J. (2017) 474:1823–36. 10.1042/BCJ20160510 28512250 PMC5433529

[3] Pickard JM Zeng MY Caruso R Núñez G. Gut microbiota: Role in pathogen colonization, immune responses, and inflammatory disease. Immunol Rev. (2017) 279:70–89. 10.1111/imr.12567 28856738 PMC5657496

[4] Kim H Sitarik AR Woodcroft K Johnson CC Zoratti E. Birth mode, breastfeeding, pet exposure, and antibiotic use: associations with the gut microbiome and sensitization in children. Curr Aller Asthma Rep. (2019) 19:1–9. 10.1007/s11882-019-0851-9 30859338 PMC7376540

[5] Francino MP. Birth mode-related differences in gut microbiota colonization and immune system development. Ann Nutr Metabol. (2018) 73:12–6. 10.1159/000490842 30041189

[6] Young VB. The intestinal microbiota in health and disease. Curr Opin Gastroenterol. (2012) 28:63. 10.1097/MOG.0b013e32834d61e9 22080827 PMC3707308

[7] Turroni F Milani C Duranti S Lugli GA Bernasconi S Margolles A. The infant gut microbiome as a microbial organ influencing host well-being. Ital J Pediatr. (2020) 46:1–3. 10.1186/s13052-020-0781-0 32024556 PMC7003403

[8] D'Argenio V. Human microbiome acquisition and bioinformatic challenges in metagenomic studies. Int J Mol Sci. (2018) 19:383. 10.3390/ijms19020383 29382070 PMC5855605

[9] Milani C Duranti S Bottacini F Casey E Turroni F Mahony J. The first microbial colonizers of the human gut: composition, activities, and health implications of the infant gut microbiota. Microbiol Molec Biol Rev. (2017) 81:e00036–17. 10.1128/MMBR.00036-17 29118049 PMC5706746

[10] Wang Q Wang K Wu W Giannoulatou E Ho JWK Li L. Host and microbiome multiomics integration: applications and methodologies. Biophys Rev. (2019) 11:55–65. 10.1007/s12551-018-0491-7 30627872 PMC6381360

[11] Abellan-Schneyder I Matchado MS Reitmeier S Sommer A Sewald Z Baumbach J. Primer, pipelines, parameters: issues in 16S rRNA gene sequencing. MSphere. (2021) 6:e01202–20. 10.1128/mSphere.01202-20 33627512 PMC8544895

[12] Perez-Muñoz ME Arrieta MC Ramer-Tait AE Walter J. A critical assessment of the “sterile womb” and “in utero colonization” hypotheses: implications for research on the pioneer infant microbiome. Microbiome. (2017) 5:1–9. 10.1186/s40168-017-0268-4 28454555 PMC5410102

[13] Aagaard K Ma J Antony KM Ganu R Petrosino J Versalovic J. The placenta harbors a unique microbiome. Sci Translat Med. (2014) 6:237ra65. 10.1126/scitranslmed.3008599 24848255 PMC4929217

[14] He Q Kwok LY Xi X Zhong Z Ma T Xu H. The meconium microbiota shares more features with the amniotic fluid microbiota than the maternal fecal and vaginal microbiota. Gut Microbes. (2020) 12:1794266. 10.1080/19490976.2020.1794266 32744162 PMC7524391

[15] Cabrera-Rubio R Mira-Pascual L Mira A Collado MC. Impact of mode of delivery on the milk microbiota composition of healthy women. J Dev Orig Health Dis. (2016) 7:54–60. 10.1017/S2040174415001397 26286040

[16] Romero R Miranda J Chaiworapongsa T Chaemsaithong P Gotsch F Dong Z. Sterile and microbial-associated intra-amniotic inflammation in preterm prelabor rupture of membranes. J Maternal-Fetal Neonatal Med. (2015) 28:1394–409. 10.3109/14767058.2014.958463 25190175 PMC5371030

[17] Wang X Buhimschi CS Temoin S Bhandari V Han YW Buhimschi IA. Comparative microbial analysis of paired amniotic fluid and cord blood from pregnancies complicated by preterm birth and early-onset neonatal sepsis. PLoS ONE. (2013) 8:e56131. 10.1371/journal.pone.0056131 23437088 PMC3577789

[18] Mesa MD Loureiro B Iglesia I Fernandez Gonzalez S Llurba Olivé E. The evolving microbiome from pregnancy to early infancy: a comprehensive review. Nutrients. (2020) 12:133. 10.3390/nu12010133 31906588 PMC7019214

[19] Olomu IN Pena-Cortes LC Long RA Vyas A Krichevskiy O Luellwitz R. Elimination of “kitome” and “splashome” contamination results in lack of detection of a unique placental microbiome. BMC Microbiol. (2020) 20:1–9. 10.1186/s12866-020-01839-y 32527226 PMC7291729

[20] Rackaityte E Halkias J Fukui EM Mendoza VF Hayzelden C Crawford ED. Viable bacterial colonization is highly limited in the human intestine in utero. Nat Med. (2020) 26:599–607. 10.1038/s41591-020-0761-3 32094926 PMC8110246

[21] Kuperman AA Zimmerman A Hamadia S Ziv O Gurevich V Fichtman B. Deep microbial analysis of multiple placentas shows no evidence for a placental microbiome. Int J Obstetr Gynaecol. (2020) 127:159–69. 10.1111/1471-0528.15896 31376240

[22] Stinson LF Keelan JA Payne MS. Identification and removal of contaminating microbial DNA from PCR reagents: impact on low-biomass microbiome analyses. Lett Appl Microbiol. (2019) 68:2–8. 10.1111/lam.13091 30383890

[23] Lauder AP Roche AM Sherrill-Mix S Bailey A Laughlin AL Bittinger K. Comparison of placenta samples with contamination controls does not provide evidence for a distinct placenta microbiota. Microbiome. (2016) 4:1–1. 10.1186/s40168-016-0172-3 27338728 PMC4917942

[24] Valdes AM Walter J Segal E Spector TD. Role of the gut microbiota in nutrition and health. BMJ. (2018) 361:36–34. 10.1136/bmj.k2179 29899036 PMC6000740

[25] Fujimura KE Slusher NA Cabana MD Lynch SV. Role of the gut microbiota in defining human health. Expert Rev Anti Infect Ther. (2010) 8:435–54. 10.1586/eri.10.14 20377338 PMC2881665

[26] Flint HJ cott KP Louis P Duncan SH. The role of the gut microbiota in nutrition and health. Nat Rev Gastroenterol Hepatol. (2012) 9:577–89. 10.1038/nrgastro.2012.156 22945443

[27] Moossavi S Sepehri S Robertson B Bode L Goruk S Field CJ. Composition and variation of the human milk microbiota are influenced by maternal and early-life factors. Cell Host Microbe. (2019) 25:324–235. 10.1016/j.chom.2019.01.011 30763539

[28] Walker WA. The importance of appropriate initial bacterial colonization of the intestine in newborn, child, and adult health. Pediatr Res. (2017) 82:387–95. 10.1038/pr.2017.111 28426649 PMC5570628

[29] Wang S Ryan CA Boyaval P Dempsey EM Ross RP Stanton C. Maternal vertical transmission affecting early-life microbiota development. Trends Microbiol. (2020) 28:28–45. 10.1016/j.tim.2019.07.010 31492538

[30] Zimmermann P Curtis N. Factors influencing the intestinal microbiome during the first year of life. Pediatr Infect Dis J. (2018) 37:e315–e335. 10.1097/INF.0000000000002103 29746379

[31] Derrien M Alvarez AS de Vos WM. The gut microbiota in the first decade of life. Trends Microbiol. (2019) 27:997–1010. 10.1016/j.tim.2019.08.001 31474424

[32] Tanaka M Nakayama J. Development of the gut microbiota in infancy and its impact on health in later life. Allergol Int. (2017) 66:515–22. 10.1016/j.alit.2017.07.010 28826938

[33] Mitchell C Hogstrom L Bryant A Bergerat A Cher A Pochan S. Delivery mode impacts newborn gut colonization efficiency. bioRxiv. (2020). 10.1101/2020.01.29.919993

[34] Chu DM Ma J Prince AL Antony KM Seferovic MD Aagaard KM. Maturation of the infant microbiome community structure and function across multiple body sites and in relation to mode of delivery. Nat Med. (2017) 23:314–26. 10.1038/nm.4272 28112736 PMC5345907

[35] Princisval L Rebelo F Williams BL Coimbra AC Crovesy L Ferreira AL. Association between the mode of delivery and infant gut microbiota composition up to 6 months of age: a systematic literature review considering the role of breastfeeding. Nutr Rev. (2021) 80:113–27. 10.1093/nutrit/nuab008 33837424

[36] Lee E Kim BJ Kang MJ Choi KY Cho HJ Kim Y. Dynamics of gut microbiota according to the delivery mode in healthy Korean infants. Allergy Asthma Immunol Res. (2016) 8:471–7. 10.4168/aair.2016.8.5.471 27334787 PMC4921703

[37] Cunnington AJ Sim K Deierl A Kroll JS Brannigan E Darby J. “Vaginal seeding” of infants born by caesarean section. BMJ. (2016) 3352:i227. 10.1136/bmj.i227 26906151

[38] Sanidad KZ Zeng MY. Neonatal gut microbiome and immunity. Curr Opin Microbiol. (2020) 56:30–7. 10.1016/j.mib.2020.05.011 32634598 PMC8729197

[39] Tirone C Pezza L Paladini A Tana M Aurilia C Lio A. Gut and lung microbiota in preterm infants: immunological modulation and implication in neonatal outcomes. Front Immunol. (2019) 10:2910. 10.3389/fimmu.2019.02910 31921169 PMC6920179

[40] Tauchi H Yahagi K Yamauchi T Hara T Yamaoka R Tsukuda N. Gut microbiota development of preterm infants hospitalised in intensive care units. Benef Microbes. (2019) 10:641–51. 10.3920/BM2019.0003 31179713

[41] Arboleya S Binetti A Salazar N Fernández N Solís G Hernandez-Barranco A. Establishment and development of intestinal microbiota in preterm neonates. FEMS Microbiol Ecol. (2012) 79:763–72. 10.1111/j.1574-6941.2011.01261.x 22126419

[42] Henderickx JGE Zwittink RD van Lingen RA Knol J Belzer C. The preterm gut microbiota: an inconspicuous challenge in nutritional neonatal care. Front Cell Infect Microbiol. (2019) 9:85. 10.3389/fcimb.2019.00085 31001489 PMC6454191

[43] Dalby MJ Hall LJ. Recent advances in understanding the neonatal microbiome. F1000Research. (2020) 9:422. 10.12688/f1000research.22355.1 32518631 PMC7255898

[44] Leggett RM Alcon-Giner C Heavens D Caim S Brook TC Kujawska M. Rapid MinION profiling of preterm microbiota and antimicrobial-resistant pathogens. Nat Microbiol. (2020) 5:430–42. 10.1038/s41564-019-0626-z 31844297 PMC7044117

[45] Khodayar-Pardo P Mira-Pascual L Collado MC Martínez-Costa C. Impact of lactation stage, gestational age and mode of delivery on breast milk microbiota. J Perinatol. (2014) 34:599–605. 10.1038/jp.2014.47 24674981

[46] Schwartz DJ Langdon AE Dantas G. Understanding the impact of antibiotic perturbation on the human microbiome. Genome Med. (2020) 12:1–2. 10.1186/s13073-020-00782-x 32988391 PMC7523053

[47] Bokulich NA Chung J Battaglia T Henderson N Jay M Li H. Antibiotics, birth mode, and diet shape microbiome maturation during early life. Sci Translat Med. (2016) 8:343ra82. 10.1126/scitranslmed.aad7121 27306664 PMC5308924

[48] Tamburini S Shen N Wu HC Clemente JC. The microbiome in early life: implications for health outcomes. Nat Med. (2016) 22:713–22. 10.1038/nm.4142 27387886

[49] Stiemsma LT Michels KB. The role of the microbiome in the developmental origins of health and disease. Pediatrics. (2018) 141:e20172437. 10.1542/peds.2017-2437 29519955 PMC5869344

[50] Seelbach-Goebel B. Antibiotic therapy for premature rupture of membranes and preterm labor and effect on fetal outcome. Geburtshilfe Frauenheilkunde. (2013) 73:1218–27. 10.1055/s-0033-1360195 24771902 PMC3964356

[51] Fernández L Langa S Martín V Maldonado A Jiménez E Martín R. The human milk microbiota: origin and potential roles in health and disease. Pharmacol Res. (2013) 69:1–10. 10.1016/j.phrs.2012.09.001 22974824

[52] Vega-Bautista A de la Garza M Carrero JC Campos-Rodríguez R Godínez-Victoria M Drago-Serrano ME. The impact of lactoferrin on the growth of intestinal inhabitant Bacteria. Int J Molec Sci. (2019) 20:4707. 10.3390/ijms20194707 31547574 PMC6801499

[53] Quigley M Embleton ND McGuire W. Formula versus donor breast milk for feeding preterm or low birth weight infants. Cochrane Datab System Rev. (2018) 6:CD002971. 10.1002/14651858.CD002971.pub4 29926476 PMC6513381

[54] Boix-Amorós A Collado MC Mira A. Relationship between milk microbiota, bacterial load, macronutrients, and human cells during lactation. Front Microbiol. (2016) 7:492. 10.3389/fmicb.2016.00492 27148183 PMC4837678

[55] Gopalakrishna KP Hand TW. Influence of maternal milk on the neonatal intestinal microbiome. Nutrients. (2020) 12:823. 10.3390/nu12030823 32244880 PMC7146310

[56] Mira A Rodríguez JM. The origin of human milk bacteria. In: McGuire M, McGuire MA, Bode L, editors. Prebiotics and Probiotics in Human Milk: Origins and Functions of Milk-Borne Oligosaccharides and Bacteria. Cambridge, MA: Academic Press (2017) p. 349–64. 10.1016/B978-0-12-802725-7.00013-0

[57] Rodríguez JM. The origin of human milk bacteria: is there a bacterial entero-mammary pathway during late pregnancy and lactation? Adv Nutr. (2014) 5:779–84. 10.3945/an.114.007229 25398740 PMC4224214

[58] Wang Z. Comparing Gut Microbiome Virome in the Breast Milk-and Formula-fed Late Preterm Infants. Electronic Theses Dissertations (2019). p. 3375. Available online at: https://openprairie.sdstate.edu/etd/3375 (accessed October 18, 2021).

[59] Gurnani M Birken C Hamilton J. Childhood obesity: causes, consequences, and management. Pediatr Clin. (2015) 62:821–40. 10.1016/j.pcl.2015.04.001 26210619

[60] Xu J Lawley B Wong G Otal A Chen L Ying TJ. Ethnic diversity in infant gut microbiota is apparent before the introduction of complementary diets. Gut Microbes. (2020) 11:1362–73. 10.1080/19490976.2020.1756150 32453615 PMC7524347

[61] Zhou S Xu R He F Zhou J Wang Y Zhou J. Diversity of Gut Microbiota Metabolic Pathways in 10 Pairs of Chinese Infant Twins. PLoS ONE. (2016) 11:e0161627. 10.1371/journal.pone.0161627 27583441 PMC5008625

[62] Daliri EB Ofosu FK Chelliah R Lee BH Oh DH. Challenges and Perspective in Integrated Multi-Omics in Gut Microbiota Studies. Biomolecules. (2021) 11:300. 10.3390/biom11020300 33671370 PMC7922017

[63] Vernocchi P Del Chierico F Putignani L. Gut microbiota profiling: metabolomics based approach to unravel compounds affecting human health. Front Microbiol. (2016) 7:1144. 10.3389/fmicb.2016.01144 27507964 PMC4960240

[64] Beck LC Granger CL Masi AC Stewart CJ. Use of omic technologies in early life gastrointestinal health and disease: from bench to bedside. Expert Rev Proteomics. (2021) 18:247–59. 10.1080/14789450.2021.1922278 33896313

[65] Zhang X Li L Butcher J Stintzi A Figeys D. Advancing functional and translational microbiome research using meta-omics approaches. Microbiome. (2019) 7:1–2. 10.1186/s40168-019-0767-6 31810497 PMC6898977

[66] Peters DL Wang W Zhang X Ning Z Mayne J Figeys D. Metaproteomic and Metabolomic Approaches for Characterizing the Gut Microbiome. Proteomics. (2019) 19:e1800363. 10.1002/pmic.201800363 31321880

[67] Bolyen E Rideout JR Dillon MR Bokulich NA Abnet CC Al-Ghalith GA. Reproducible, interactive, scalable and extensible microbiome data science using QIIME2. Nat Biotechnol. (2019) 37:852–7. 10.1038/s41587-019-0209-9 31341288 PMC7015180

[68] Schloss PD Westcott SL Ryabin T Hall JR Hartmann M Hollister EB. Introducing mothur: open-source, platform-independent, community-supported software for describing and comparing microbial communities. Appl Environ Microbiol. (2009) 75:7537–41. 10.1128/AEM.01541-09 19801464 PMC2786419

[69] Callahan BJ McMurdie PJ Rosen MJ Han AW Johnson AJ Holmes SP. DADA2: High-resolution sample inference from Illumina amplicon data. Nat Methods. (2016) 13:581–3. 10.1038/nmeth.3869 27214047 PMC4927377

[70] Eren AM Morrison HG Lescault PJ Reveillaud J Vineis JH Sogin ML. Minimum entropy decomposition: unsupervised oligotyping for sensitive partitioning of high-throughput marker gene sequences. ISME J. (2015) 9:968–79. 10.1038/ismej.2014.195 25325381 PMC4817710

[71] Edgar RC. UNOISE2: improved error-correction for Illumina 16S and ITS amplicon sequencing. BioRxiv. (2016) 081257. 10.1101/081257

[72] Moossavi S Atakora F Fehr K Khafipour E. Biological observations in microbiota analysis are robust to the choice of 16S rRNA gene sequencing processing algorithm: case study on human milk microbiota. BMC Microbiol. (2020) 20:1–9. 10.1186/s12866-020-01949-7 32948144 PMC7501722

[73] Bharti R Grimm DG. Current challenges and best-practice protocols for microbiome analysis. Brief Bioinform. (2021) 22:178–93. 10.1093/bib/bbz155 31848574 PMC7820839

[74] Callahan BJ McMurdie PJ Holmes SP. Exact sequence variants should replace operational taxonomic units in marker-gene data analysis. ISME J. (2017) 11:2639–43. 10.1038/ismej.2017.119 28731476 PMC5702726

[75] Pérez-Cobas AE Gomez-Valero L Buchrieser C. Metagenomic approaches in microbial ecology: an update on whole-genome and marker gene sequencing analyses. Microbial Genom. (2020) 6:8. 10.1099/mgen.0.000409 32706331 PMC7641418

[76] Prodan A Tremaroli V Brolin H Zwinderman AH Nieuwdorp M Levin E. Comparing bioinformatic pipelines for microbial 16S rRNA amplicon sequencing. PLoS ONE. (2020) 15:e0227434. 10.1371/journal.pone.0227434 31945086 PMC6964864

[77] Marizzoni M Gurry T Provasi S Greub G Lopizzo N Ribaldi F. Comparison of bioinformatics pipelines and operating systems for the analyses of 16S rRNA gene amplicon sequences in human fecal samples. Front Microbiol. (2020) 11:1262. 10.3389/fmicb.2020.01262 32636817 PMC7318847

[78] Matias Rodrigues JF Schmidt TSB Tackmann J von Mering C. MAPseq: highly efficient k-mer search with confidence estimates, for rRNA sequence analysis. Bioinformatics. (2017) 33:3808–10. 10.1093/bioinformatics/btx517 28961926 PMC5860325

[79] Balvočiute M Huson DH. SILVA, RDP, Greengenes, NCBI and OTT - how do these taxonomies compare? BMC Genomics. (2017) 18:1–8. 10.1186/s12864-017-3501-4 28361695 PMC5374703

[80] Kennedy KM Gerlach MJ Adam T Heimesaat MM Rossi L Surette MG. Fetal meconium does not have a detectable microbiota before birth. Nature Microbiology. (2021) 6:1–9. 10.1101/2021.02.17.431710 33972766

[81] Li N Liang S Chen Q Zhao L Li B Huo G. Distinct gut microbiota and metabolite profiles induced by delivery mode in healthy Chinese infants. J Proteomics. (2021) 232:104071. 10.1016/j.jprot.2020.104071 33307251

[82] Dinleyici M Pérez-Brocal V Arslanoglu S Aydemir O Sevuk Ozumut S Tekin N. Human milk virome analysis: changing pattern regarding mode of delivery, birth weight, and lactational stage. Nutrients. (2021) 13:1779. 10.3390/nu13061779 34071061 PMC8224552

[83] Toivonen L Schuez-Havupalo L Karppinen S Waris M Hoffman KL Camargo CA. Antibiotic treatments during infancy, changes in nasal microbiota, and asthma development: population-based cohort study. Clin Infect Dis. (2021) 72:1546–54. 10.1093/cid/ciaa262 32170305 PMC8096219

[84] Gong C Yang L Liu K Shen S Zhang Q Li H. Effects of Antibiotic Treatment and Probiotics on the Gut Microbiome of 40 Infants Delivered Before Term by Cesarean Section Analysed by Using 16S rRNA Quantitative Polymerase Chain Reaction Sequencing. Int Med J Exper Clin Res. (2021) 27:e928467–1. 10.12659/MSM.928467 33542172 PMC7871509

[85] Selma-Royo M García-Mantrana I Calatayud M Parra-Llorca A Martínez-Costa C Collado MC. Maternal diet during pregnancy and intestinal markers are associated with early gut microbiota. J Nutr. (2021) 60:1429–42. 10.1007/s00394-020-02337-7 32728880

[86] Wood H Acharjee A Pearce H Quraishi MN Powell R Rossiter A. Breastfeeding promotes early neonatal regulatory T-cell expansion and immune tolerance of non-inherited maternal antigens. Allergy. (2021) 76:2447–60. 10.1111/all.14736 33432577

[87] Chin N Méndez-Lagares G Taft DH Laleau V Kieu H Narayan NR. Transient effect of infant formula supplementation on the intestinal microbiota. Nutrients. (2021) 13:807. 10.3390/nu13030807 33804415 PMC7998963

[88] Cortes-Macías E Selma-Royo M García-Mantrana I Calatayud M González S Martínez-Costa C. Maternal diet shapes the breast milk microbiota composition and diversity: impact of mode of delivery and antibiotics exposure. J Nutr. (2021) 151:330–40. 10.1093/jn/nxaa310 33188413 PMC7850106

[89] Mueller NT Differding MK Østbye T Hoyo C Benjamin-Neelon SE. Association of birth mode of delivery with infant faecal microbiota, potential pathobionts, and short chain fatty acids: a longitudinal study over the first year of life. Int J Obstetr Gynaecol. (2021) 128:1293–303. 10.1111/1471-0528.16633 33338292 PMC8211907

[90] Morais J Marques C Faria A Teixeira D Barreiros-Mota I Durão C. Influence of human milk on very preterms' gut microbiota and alkaline phosphatase activity. Nutrients. (2021) 13:1564. 10.3390/nu13051564 34066473 PMC8148101

[91] Pan K Zhang C Tian J. The effects of different modes of delivery on the structure and predicted function of intestinal microbiota in neonates and early infants. Polish J Microbiol. (2021) 70:45–55. 10.33073/pjm-2021-002 33815526 PMC8008759

[92] Fan HY Tung YT Yang YC Hsu JB Lee CY Chang TH. Maternal vegetable and fruit consumption during pregnancy and its effects on infant gut microbiome. Nutrients. (2021) 13:1559. 10.3390/nu13051559 34063157 PMC8148194

[93] Marrs T Jo JH Perkin MR Rivett DW Witney AA Bruce KD. Gut microbiota development during infancy: Impact of introducing allergenic foods. J Allergy Clin Immunol. (2021) 147:613–2. 10.1016/j.jaci.2020.09.042 33551026 PMC9169695

[94] Didelot X Walker AS Peto TE Crook DW Wilson DJ. Within-host evolution of bacterial pathogens. Nat Rev Microbiol. (2016) 14:150–62. 10.1038/nrmicro.2015.13 26806595 PMC5053366

[95] Sharpton TJ. An introduction to the analysis of shotgun metagenomic data. Front Plant Sci. (2014) 5:209. 10.3389/fpls.2014.00209 24982662 PMC4059276

[96] Oulas A Pavloudi C Polymenakou P Pavlopoulos GA Papanikolaou N et al. Metagenomics: tools and insights for analyzing next-generation sequencing data derived from biodiversity studies. Bioinform Biol Insights. (2015) 9:BBI-S12462. 10.4137/BBI.S12462 25983555 PMC4426941

[97] Segal JP Mullish BH Quraishi MN Acharjee A Williams HRT et al. The application of omics techniques to understand the role of the gut microbiota in inflammatory bowel disease. Therap Adv Gastroenterol. (2019) 12:1756284818822250. 10.1177/1756284818822250 30719076 PMC6348496

[98] Tang ZZ Chen G Hong Q Huang S Smith HM Shah RD. Multi-omic analysis of the microbiome and metabolome in healthy subjects reveals microbiome-dependent relationships between diet and metabolites. Front Genet. (2019) 10:454. 10.3389/fgene.2019.00454 31164901 PMC6534069

[99] Pettersen VK Antunes LCM Dufour A Arrieta MC. Inferring early-life host and microbiome functions by mass spectrometry-based metaproteomics and metabolomics. Comput Struct Biotechnol J. (2021) 20:274–286. 10.1016/j.csbj.2021.12.012 35024099 PMC8718658

[100] Cambiaghi A Ferrario M Masseroli M. Analysis of metabolomic data: tools, current strategies and future challenges for omics data integration. Briefings Bioinform. (2017) 18:498–510. 10.1093/bib/bbw031 27075479

[101] Selway CA Eisenhofer R Weyrich LS. Microbiome applications for pathology: challenges of low microbial biomass samples during diagnostic testing. J Pathol Clin Res. (2020) 6:97–106. 10.1002/cjp2.151 31944633 PMC7164373

[102] Eisenhofer R Minich JJ Marotz C Cooper A Knight R Weyrich LS. Contamination in low microbial biomass microbiome studies: issues and recommendations. Trends Microbiol. (2019) 27:105–17. 10.1016/j.tim.2018.11.003 30497919

[103] Minich JJ Zhu Q Janssen S Hendrickson R Amir A Vetter R. KatharoSeq Enables High-Throughput Microbiome Analysis from Low-Biomass Samples. mSystems. (2018) 3:e00218–17. 10.1128/mSystems.00218-17 29577086 PMC5864415

[104] Zinter MS Mayday MY Ryckman KK Jelliffe-Pawlowski LL DeRisi JL. Towards precision quantification of contamination in metagenomic sequencing experiments. Microbiome. (2019) 7:1–5. 10.1186/s40168-019-0678-6 30992055 PMC6469116

[105] Chafee M Maignien L Simmons SL. The effects of variable sample biomass on comparative metagenomics. Environ Microbiol. (2015) 17:2239–53. 10.1111/1462-2920.12668 25329041

[106] Davis NM Proctor DM Holmes SP Relman DA Callahan BJ. Simple statistical identification and removal of contaminant sequences in marker-gene and metagenomics data. Microbiome. (2018) 6:1–4. 10.1186/s40168-018-0605-2 30558668 PMC6298009

